# Aldehyde dehydrogenase inhibition combined with phenformin treatment reversed NSCLC through ATP depletion

**DOI:** 10.18632/oncotarget.10354

**Published:** 2016-06-30

**Authors:** Joon Hee Kang, Seon-Hyeong Lee, Jae-Seon Lee, Boas Nam, Tae Wha Seong, Jaekyoung Son, Hyonchol Jang, Kyeong Man Hong, Cheolju Lee, Soo-Youl Kim

**Affiliations:** ^1^ Cancer Cell and Molecular Biology Branch, Research Institute, National Cancer Center, Goyang, Gyeonggi-do 410-769, Republic of Korea; ^2^ Department of Biomedical Sciences, University of Ulsan College of Medicine, Seoul 138-736, Republic of Korea; ^3^ Center for Theragnosis, Biomedical Research Institute, Korea Institute of Science and Technology, Seoul 136-791, Republic of Korea; ^4^ Department of Biological Chemistry, University of Science and Technology, Daejeon 305-333, Republic of Korea

**Keywords:** aldehyde dehydrogenase, NSCLC, gossypol, phenformin, cancer metabolism

## Abstract

Among ALDH isoforms, ALDH1L1 in the folate pathway showed highly increased expression in non-small-cell lung cancer cells (NSCLC). Based on the basic mechanism of ALDH converting aldehyde to carboxylic acid with by-product NADH, we suggested that ALDH1L1 may contribute to ATP production using NADH through oxidative phosphorylation. ALDH1L1 knockdown reduced ATP production by up to 60% concomitantly with decrease of NADH in NSCLC. ALDH inhibitor, gossypol, also reduced ATP production in a dose dependent manner together with decrease of NADH level in NSCLC. A combination treatment of gossypol with phenformin, mitochondrial complex I inhibitor, synergized ATP depletion, which efficiently induced cell death. Pre-clinical xenograft model using human NSCLC demonstrated a remarkable therapeutic response to the combined treatment of gossypol and phenformin.

## INTRODUCTION

Although many targeted therapies improved survival of lung cancer, relapse less than 14 months is the most key issue due to drug resistance [1, 2]. Therefore, understanding the metabolic character of non-small-cell lung cancer cell (NSCLC) may provide a chance to improve therapeutic approaches to drug resistance. In normal tissue, ATP is generally produced from glucose by the TCA cycle through oxidative phosphorylation in the mitochondria while cancer cells consume glucose for building anabolic precursors including nucleotide, amino acid and triglyceride [3]. Cancer cells need alternative energy sources from the microenvironment to maintain a steady state of energy. Although glycolysis is known as a major ATP supplier in mature multi-cellular tumor spheroids, glycolysis inhibition is not effective on inhibiting tumor proliferation [4]. This suggests that cells inside spheroids adopt the alternative ATP supply derived from mitochondria. However, the alternative energy source of cancer cell metabolism remains unclear.

By bioinformatics analysis of metabolic enzymes in NSCLC, we found that aldehyde dehydrogenase isoforms (ALDH) were highly increased in NSCLC (a paper under review). Among them, ALDH1L1 level in folate pathway is significantly increased in NSCLC [5, 6]. Survival analysis revealed that ALDH1L1-positive patients had a shorter overall survival rate compared with ALDH1L1-negative patients [5, 6]. However, the specific role of ALDH in the growth and survival in NSCLC remains unclear while ALDH-positive tumors are considered to be malignant. As a result of the catalytic reaction by ALDH1L1, NADH is yielded as a by-product from the conversion of 10-formyltetrahydrofolate to carbamate, which turns into 2.5 ATP through oxidative phosphorylation [7]. Therefore we proposed that NADH produced by ALDH may be a critical energy supplier in NSCLC. In this study, it is tested whether knock down of ALDH1L1 using siRNA or ALDH inhibition using gossypol may induce remarkable reduction of ATP production in NSCLC. ALDH inhibitor gossypol is employed although gossypol is characterized as pan-ALDH inhibitor, because specific inhibitor against ALDH1L1 is not available. Further it is tested whether combination of gossypol and phenformin may synergize to induce cell death through significant ATP depletion.

## RESULTS

### ALDH significantly contributes to ATP synthesis through cytosol NADH production in NSCLC

**Figure 1:**
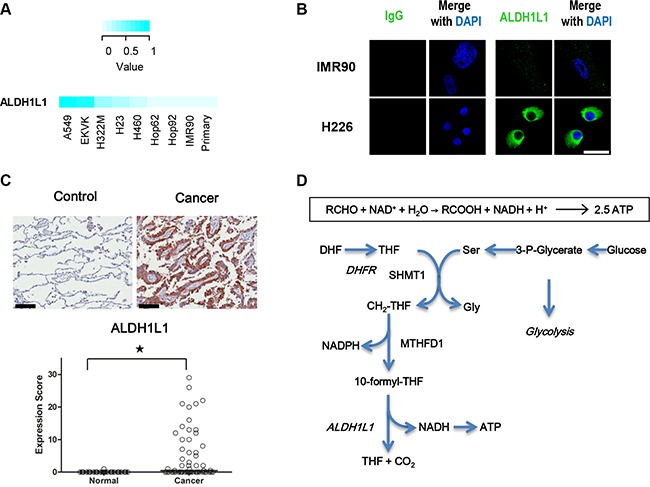
ALDH1L1 is highly increased in NSCLC (**A**) Expression of ALDH1L1 in lung cancer cell lines was measured by multiple reaction monitoring mass spectrometry (MRM-MS). (**B**) Assessment of ALDH1L1 in IMR90 and H226 by immunofluorescence staining. Scale bar = 50 μm. (**C**) Representative immunohistochemical staining of ALDH1L1 in normal and cancerous lung tissue. Scale bar = 100 μm. Expression of ALDH1L1in cancerous (Cancer) and normal lung type I and II pneumocytes (Control). *p < 0.001, *n* = 57 for each case. (**D**) ALDH1L1 catalyses 10-formyltetrahydrofolate to THF with by-product of NADH in the serine-folate pathway. DHF, dihydrofolate; THF, tetrahydrofolate; SHMT1, serine hydroxymethyltransferase 1; DHFR, dihydrofolate reductase; CH_2_-THF, 5,10-Methylenetetrahydrofolate; MTHFD1, methylenetetrahydrofolate dehydrogenase1.

To examine their differential expression accurately in NSCLC cells, the expression level of ALDH1L1 in lung cancer cell lines was measured by liquid chromatography multiple reaction monitoring mass spectrometry (MRM-MS) (Figure 1A). To compare the expression level of ALDH1L1 in the different cell lines, MRM peak areas in NSCLC were higher than normal lung epithelial cells or immortalized IMR-90 cells (Figure 1A). Immuno cytochemistry of ALDH1L1 also confirmed higher expression in NSCLC than normal immortalized IMR-90 (Figure 1B). By immunohistochmical staining analysis of ALDH1L1 using tissue microarray (59 cases each), representative cases showing strong, moderate, weak, and negative ALDH1L1 immunostaining were in Figure S1. The expression level of ALDH1L1 was significantly higher (*P* < 0.001, marked by *) in cancer tissues (cancer) than normal lung type I and II pneumocytes (normal) (Figure 1C). We hypothesized that common role of ALDH in NSCLC may be by-production of NADH by the conversion of aldehyde to carboxylic acid. ALDH1L1 in folate metabolism catalyses 10-formyl-THF to produce THF, CO_2_ and NADH (Figure 1D).

**Figure 2:**
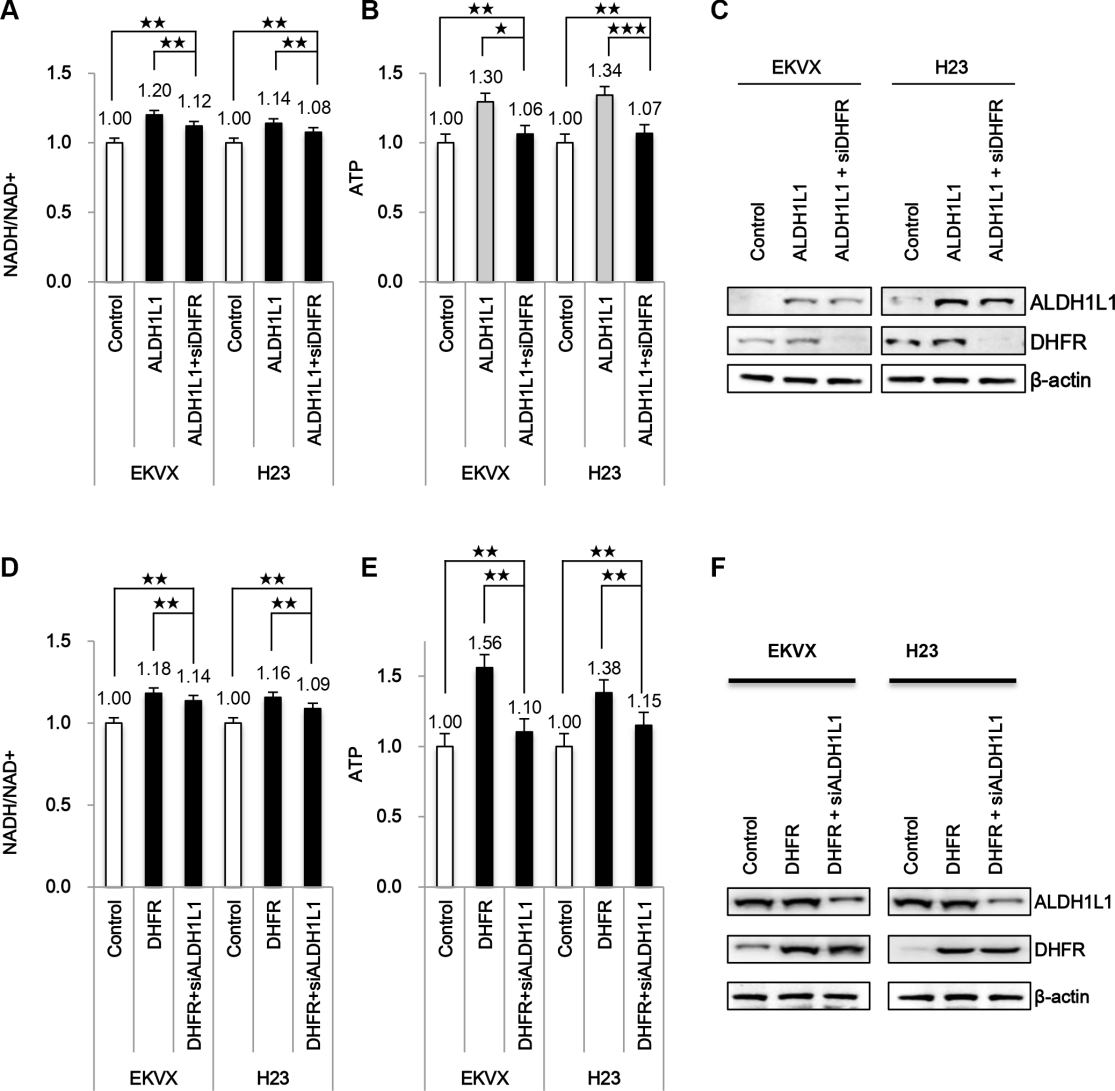
Effect of ALDH1L1 over expression on induction of NADH and ATP production (**A**–**C**) EKVX and H23 cells were transfected with plasmid expressing ALDH1L1 for 24 h and incubated with siRNA of DHFR for 24 h. (**D**–**F**) EKVX and H23 cells were transfected with plasmid expressing DHFR for 24 h and incubated with siRNA of ALDH1L1 for 24 h. Effect of DHFR siRNA or ALDH1L1 siRNA on NADH/NAD+ (A, D) and ATP (B, E) was analysed. Immunoblot analysis was performed to confirm DHFR expression, ALDH1L1 knockdown (C, F). Data are representative of the mean and standard deviation three independent experiments. *p < 0.05, **p < 0.01, ***p < 0.001 compared to vehicle control.

Thus, we tested whether NADH production by ALDH1L1 contributes to ATP production in NSCLC. Transfection of ALDH1L1 into EKVX and H23 cells increased NADH and ATP approximately 10–20% and 30%, respectively, in both cell lines (Figure 2A–2C). This increase was disrupted by blocking folate metabolism by treatment of dihydrofolate reductase (DHFR) siRNA. To test whether increased NADH and ATP is dependent on folate metabolism, the effect of DHFR overexpression was also tested. Our data demonstrate that DHFR transient transfection increased NADH and ATP 16–18% and 30–50%, respectively in EKVX and H23 (Figure 2D–2F). This increase was reversed by ALDH1L1 knockdown using siRNA.

### ATP production in NSCLC depends on the malate-aspartate shuttle for transferring cytosol NADH into mitochondria

**Figure 3:**
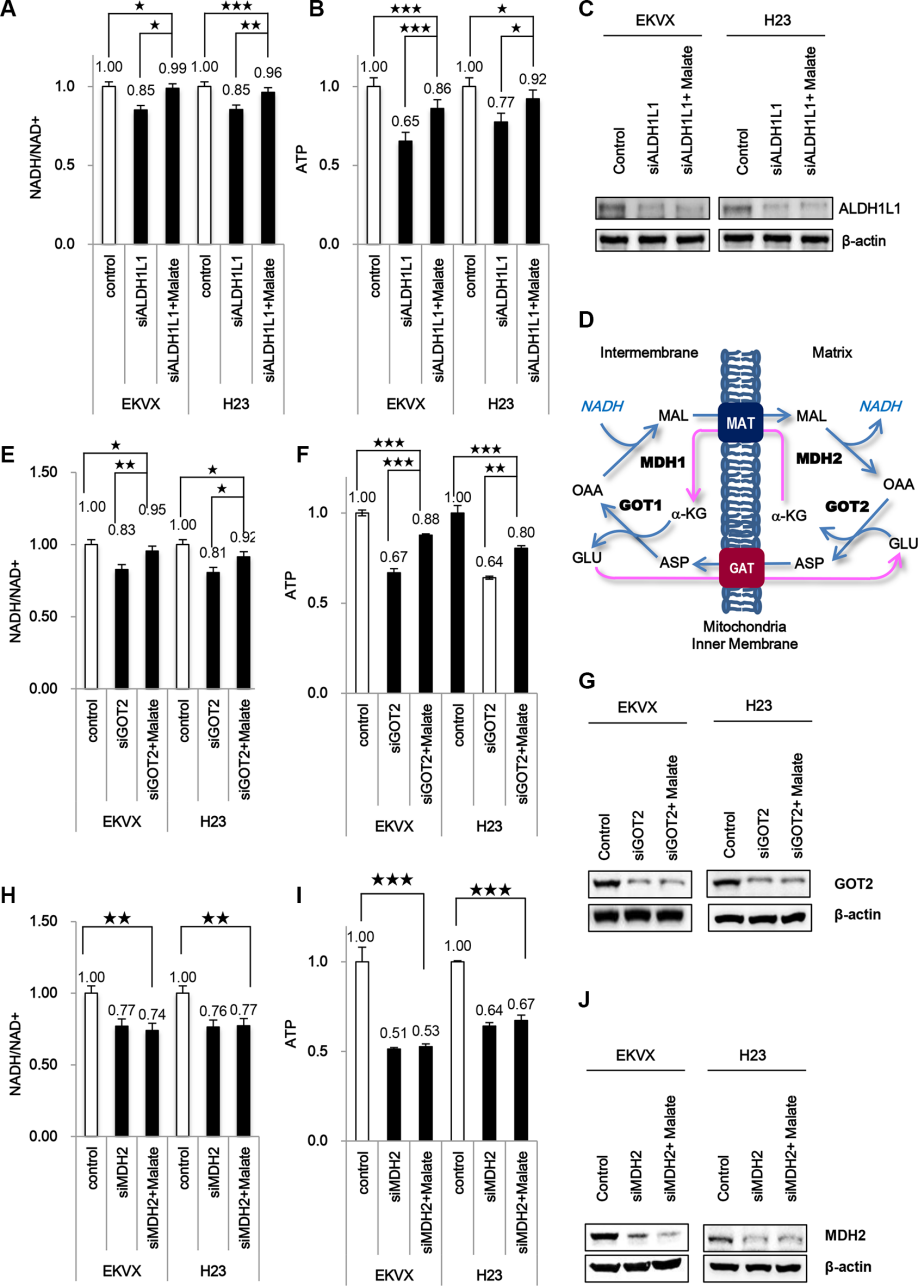
ALDH participates significantly in ATP synthesis using NADH through oxidative phosphorylation that requires the malate-aspartate shuttle in NSCLC (**A**–**C**) Effect of ALDH1L1 knockdown in the presence and absence of 10 mM malate on NADH/NAD+ (A) and ATP (B) was analysed. (**D**) Malate-aspartate shuttle for NADH transportation into the mitochondrial matrix. MAT, malate-α-ketoglutarate transporter; GAT, glutamate-aspartate transporter; OAA, oxaloacetate; α-KG, α-ketoglutarate. (**E**–**G**) Effect of GOT2 knockdown in the presence and absence of 10 mM malate on NADH/NAD+ (E) and ATP (F) was analysed. (**H**–**J**) Effect of MDH2 knockdown in the presence and absence of 10 mM malate on NADH/NAD+ (H) and ATP (I) was analysed. Immunoblot analysis was performed to confirm knockdowns of ALDH1L1, MDH2, GOT2 (C, G, J). Data are representative of the mean and standard deviation three independent experiments. *p < 0.05, **p < 0.01, ***p < 0.001 compared to vehicle control.

ALDH1L1 siRNA treatment decreased NADH and ATP by approximately 15 % and 35 %, respectively, in EKVX cells (Figure 3A–3C). NADH produced by ALDH contributes significantly to ATP synthesis in NSCLC. NADH produced by ALDH in cytosol needs to transfer into mitochondria through the malate-aspartate shuttle for electron transfer to ATP (Figure 3D). Briefly, oxaloacetate is converted to malate by malate dehydrogenase (MDH1) with oxidization of NADH to NAD in the cytoplasm. The malate enters the mitochondria by the oxoglutarate carrier as an ion transaporter (SLC25A11) [8] that must also transport α-ketoglutarate in the opposite direction by aspartate/glutamate carrier (SLC25A12) [9]. The malate is then oxidized to oxaloacetate by the mitochondrial malate dehydrogenase (MDH2), resulting in formation of NADH, which can then enter the electron transport pathway. Return of the oxaloacetate to the cytoplasm was mediated through aspartate transamination by aspartate aminotransferase (GOT2). This aspartate and α-ketoglutarate return back into the cytosol, which is then converted back to oxaloacetate and glutamate, respectively by aspartate aminotransferase (GOT1). To test whether ATP production can be decreased following inhibition of the malate-aspartate shuttle, we measured NADH and ATP in H23 and EKVX cells incubated with siRNA targeting GOT2 or MDH2 (Figure 3E–3J). Our results show that the NADH level decreased approximately 20% following incubation with GOT2 siRNA (Figure 3E, 3G). Moreover, NADH production decreased approximately 30% after MDH2 siRNA treatment (Figure 3H, 3J). The ATP level also decreased 35% in the presence of GOT2 siRNA (Figure 3F), and was reduced by 50% with MDH2 knockdown (Figure 3I). Addition of a malate supplement reversed the effect of GOT2 siRNA (Figure 3E–3G), but not MDH2 siRNA (Figure 3H–3J).

ALDH1L1 produces NADH in the cytosol which needs to be transferred into mitochondria for electron transfer from NADH to ATP. Mitochondria requires the malate-aspartate shuttle system, which transports reduced equivalents of NADH from the cytosol to mitochondria as a form of malate. To summarize the data, NSCLC remarkably depends on cytosolic NADH for ATP production, in which ALDH1L1 plays a key role for NADH production.

### ALDH1L1 knock down combined with phenformin has a significant synergistic effect on ATP reduction

**Figure 4:**
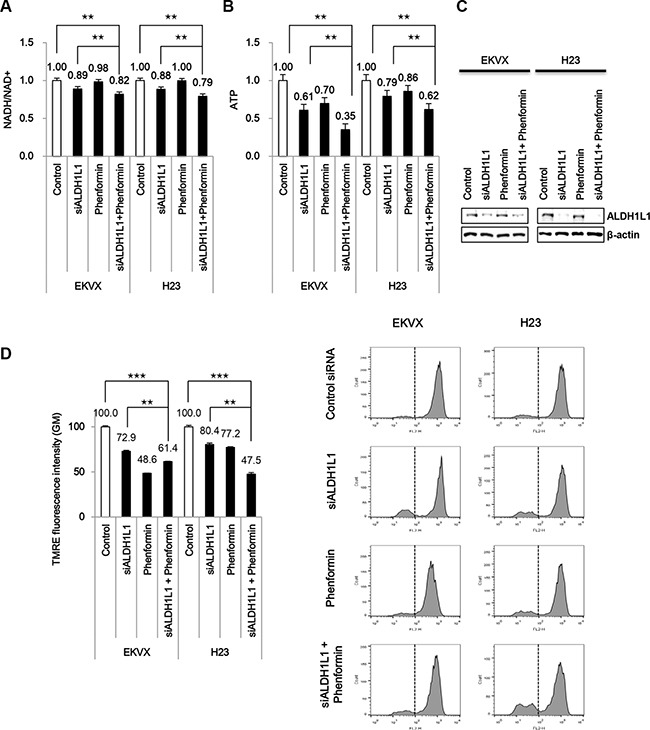
Phenformin treatment combined with siRNA of ALDH1L1 synergistically reduced ATP production (**A**, **B**) After incubation with ALDH1L1 siRNA, cells were treated with 100 μM phenformin for 24 h before assessment of NADH/NAD+ (A), ATP (B). (**C**) Immunoblot analysis was performed to confirm ALDH1L1 knockdown. (**D**) Tetramethylrhodamine ethyl ester (TMRE) staining was performed to examine changes in mitochondrial membrane potential. Data are representative of the mean and standard deviation of three independent experiments. **p < 0.01, ***p < 0.001 compared to vehicle control.

NADH is transferred to mitochondria via the malate-aspartate shuttle and donates electrons directly to the respiratory chain to convert NADH into ATP. ALDH1L1 knockdown alone using siRNA reduced NADH and ATP production by 12% and 30–40%, respectively, in EKVX and H23 (Figure 4A–4C). Phenformin has an effect on mitochondria complex I inhibition which causes decrease of ATP production resulting in AMPK activation and lower mTOR activity [10]. We tested whether ALDH1L1 knockdown combined with phenformin has a synergistic effect on ATP reduction. ALDH1L1 knockdown combined with phenformin treatment further reduced ATP production by 65% in EKVX cells (Figure 4B). To examine whether this observed decrease in ATP production is related to changes in mitochondrial membrane potential, we stained the cells for tetramethylrhodamine ethyl ester (TMRE). This assay demonstrated the collapse of the mitochondrial membrane potential (Δψm) in cells treated with ALDH1L1 siRNA, phenformin, or both (Figure 4D).

### ALDH inhibitor gossypol combined with phenformin has a significant synergistic effect on ATP reduction

**Figure 5:**
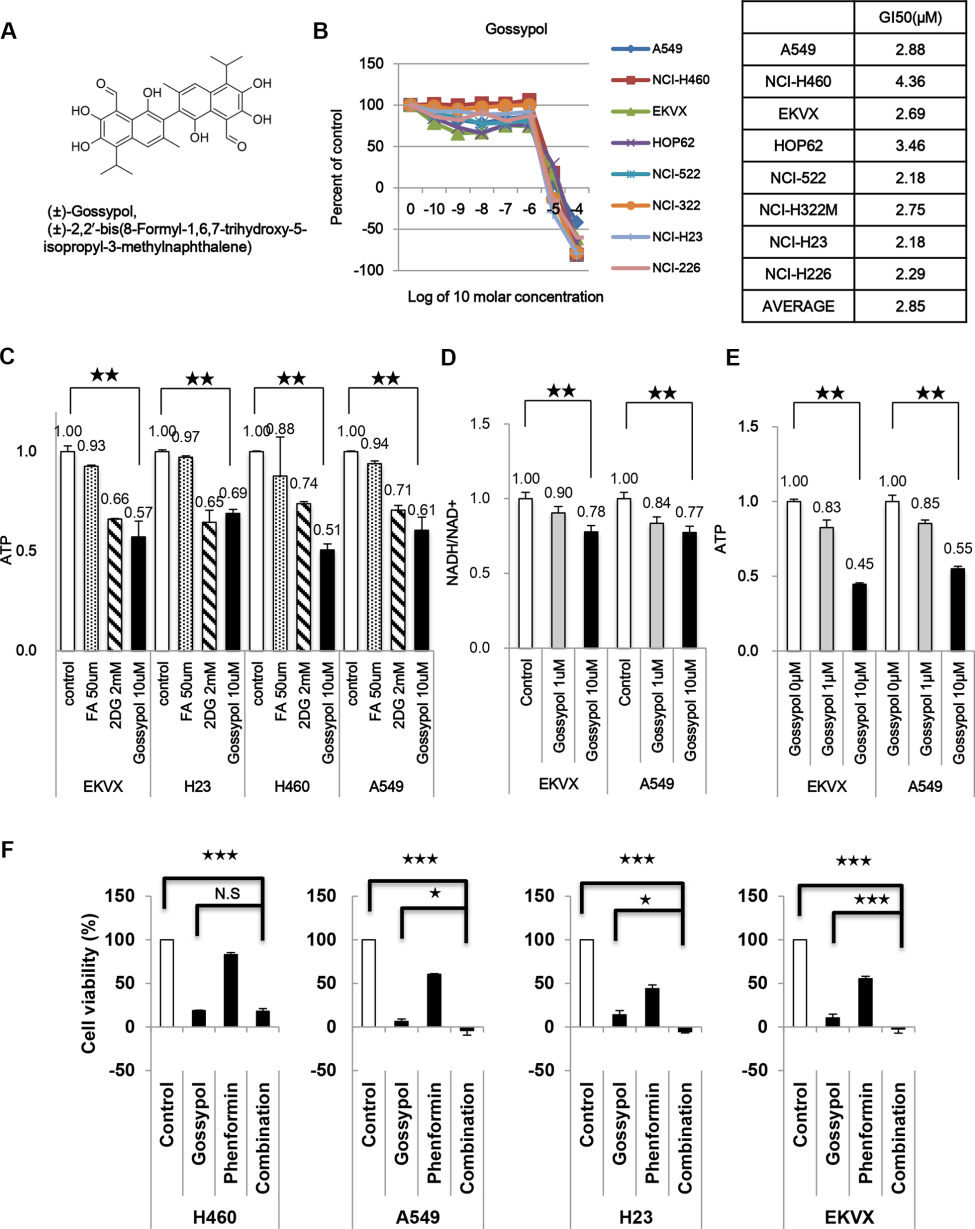
Gossypol inhibits NSCLC cell growth by decreasing ATP production (**A**) Structure of gossypol. MW = 518.563. (**B**) Effect of gossypol on NSCLC cell proliferation as determined by the SRB assay. (**C**) Cells were treated with the indicated inhibitors and then the level of ATP was determined. (**D**, **E**) The levels of NADH/NAD+ (D) and ATP (E) were measured after EKVX and A549 cells were treated with 1 and 10 μM gossypol for 24 h. (**F**) Combined treatment of 10 μM of gossypol with 100 μM phenformin showed synergistic inhibition of cell growth in NSCLC, as determined by the SRB assay. Data are representative of the mean and standard deviation of three independent experiments. *p < 0.05, **p < 0.01, ***p < 0.001 compared to vehicle control.

Pharmacological inhibitors targeting the active site have been developed for only 3 out of 19 isozymes, namely ALDH2, ALDH1A1, and ALDH3A1 [11]. Nevertheless, a natural compound called gossypol (Figure 5A) has been reported to inhibit ALDH by interacting with the cofactor binding site, suggesting that it may be a pan-ALDH inhibitor [12]. Gossypol inhibited the growth of various NSCLC, as determined by the SRB assay (average GI_50_ = 2.85 μM) (Figure 5B). To examine how ALDH inhibition reduces ATP production in glycolysis, TCA cycle and ALDH, selective inhibitors were employed for blocking glycolysis (2-DG, 2-deoxyglucose), the TCA cycle (FA, fluoroacetate, aconitase 2 inhibitor), and ALDH (gossypol) in EKVX, H23, H460, and A549 cells. Our data demonstrate that ATP production in NSCLC was significantly affected by ALDH inhibition, which reduced total ATP by approximately 35–50% (Figure 5C). Treatment of cells with 1 mM or 10 mM gossypol inhibited NADH production by approximately 10 % and 20 %, respectively (Figure 5D). Furthermore, ATP production was inhibited 25 % and 55 % following treatment with 1 mM and 10 mM gossypol, respectively (Figure 5E). Combined treatment of 10 μM of gossypol with 100 μM phenformin showed synergistic inhibition of cell growth assayed with SRB test in NSCLC (Figure 5F). Therefore, ALDH inhibition is the greatest effect on decrease of energy metabolism in cancer. To test whether any ALDH inhibitor may have the similar effect on NSCLC growth control via regulation of ATP production. ALDH specific inhibitors including daidzin and disulfiram were selected for testing SRB analysis and TMRE/FACS analysis. Daidzin is a reversible competitive inhibitor targeting ALDH2 (IC50, 80 nM) [13] while disulfiram is an irreversible inhibitor against ALDH2 and ALDH1A1 through attacking active site cysteine (IC50, 1.45 uM and 0.15 uM respectively) [14]. Cell growth was not affected by both inhibitors as well as mitochondrial action potential was not changed by both inhibitors (Figure S2). This result implies that ALDH2 or ALDH1A1 does not contribute to NSCLC cell growth through increase of ATP production. Additionally, disulfiram and daidzin do not inhibit ALDH1L1 as much as gossypol does.

### Treatment with gossypol and phenformin decreased ATP production significantly in NSCLC

**Figure 6:**
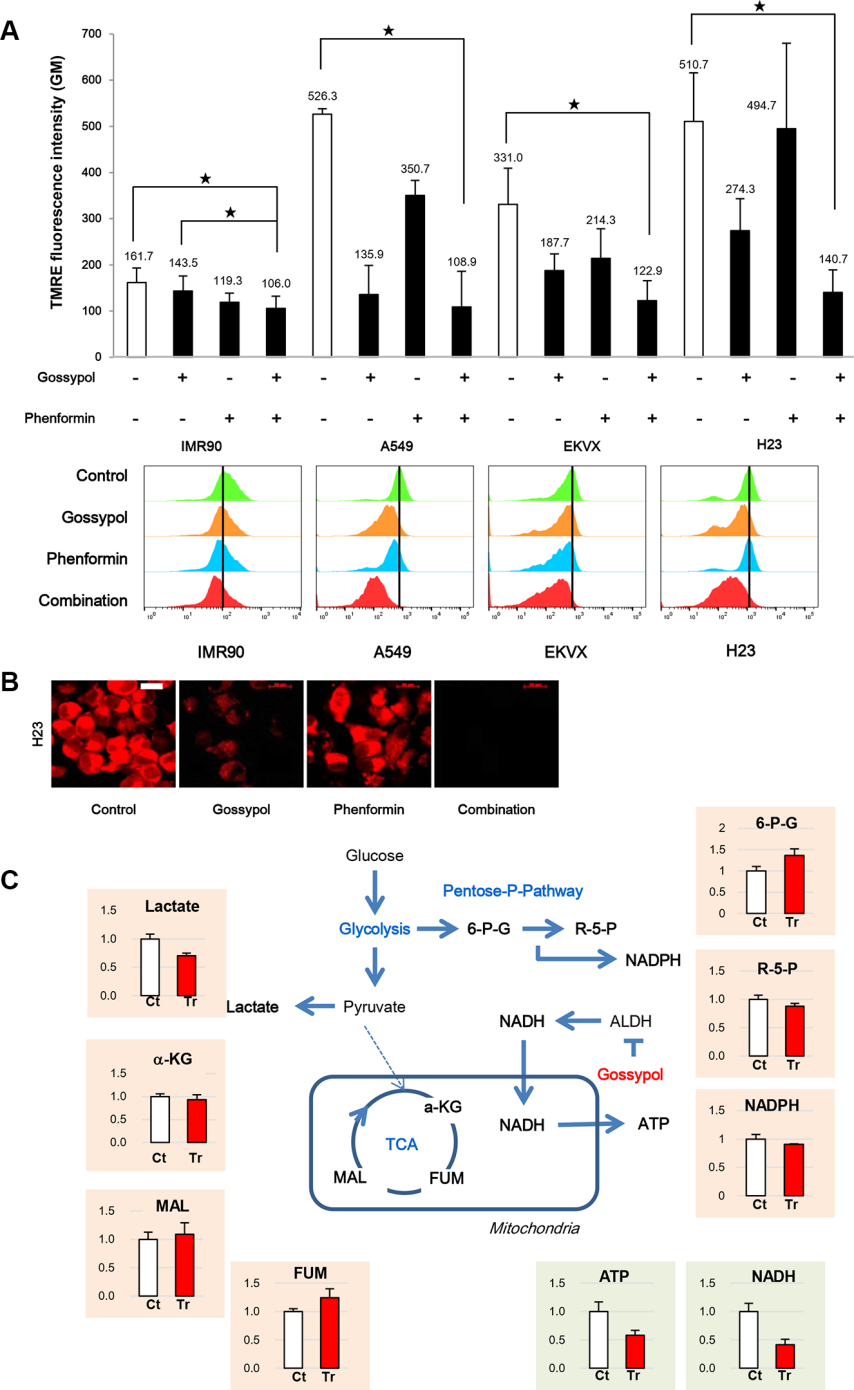
Combination of gossypol and phenformin remarkably reduced NADH and ATP production with down regulation of mitochondrial action potential (**A**) Cells were treated as indicated for 24 h, stained for TMRE, and analysed by flow cytometry and live cell imaging. Data are representative of the mean and standard deviation three independent experiments. (**B**) H23 was stained for TMRE after 24 h treatment of gossypol, phenformin, and combination. Scale bar = 20 μm. (**C**) Effect of gossypol treatment on metabolites from various metabolic pathways in A549 cells. Relative pool sizes of metabolites by targeted LC-MS/MS upon gossypol treatment for 24 h. Data are representative of the mean and standard deviation three independent experiments. *p < 0.05 compared to vehicle control.

Interestingly, the combination treatment of gossypol and phenformin reduced TMRE fluorescence by 80% and 73% in A549 and H23 cells, respectively, compared to the non-treated control (Figure 6A). The TMRE levels by combination treatment of gossypol and phenformin are reduced to the level of IMR90 cells. The fluorescence image of TMRE followed by drug treatment was taken in H23 (Figure 6B). The combination of gossypol and phenformin abolished the mitochondrial activity although gossypol alone showed significant reduction of mitochondrial activity (Figure 6B).

**Figure 7:**
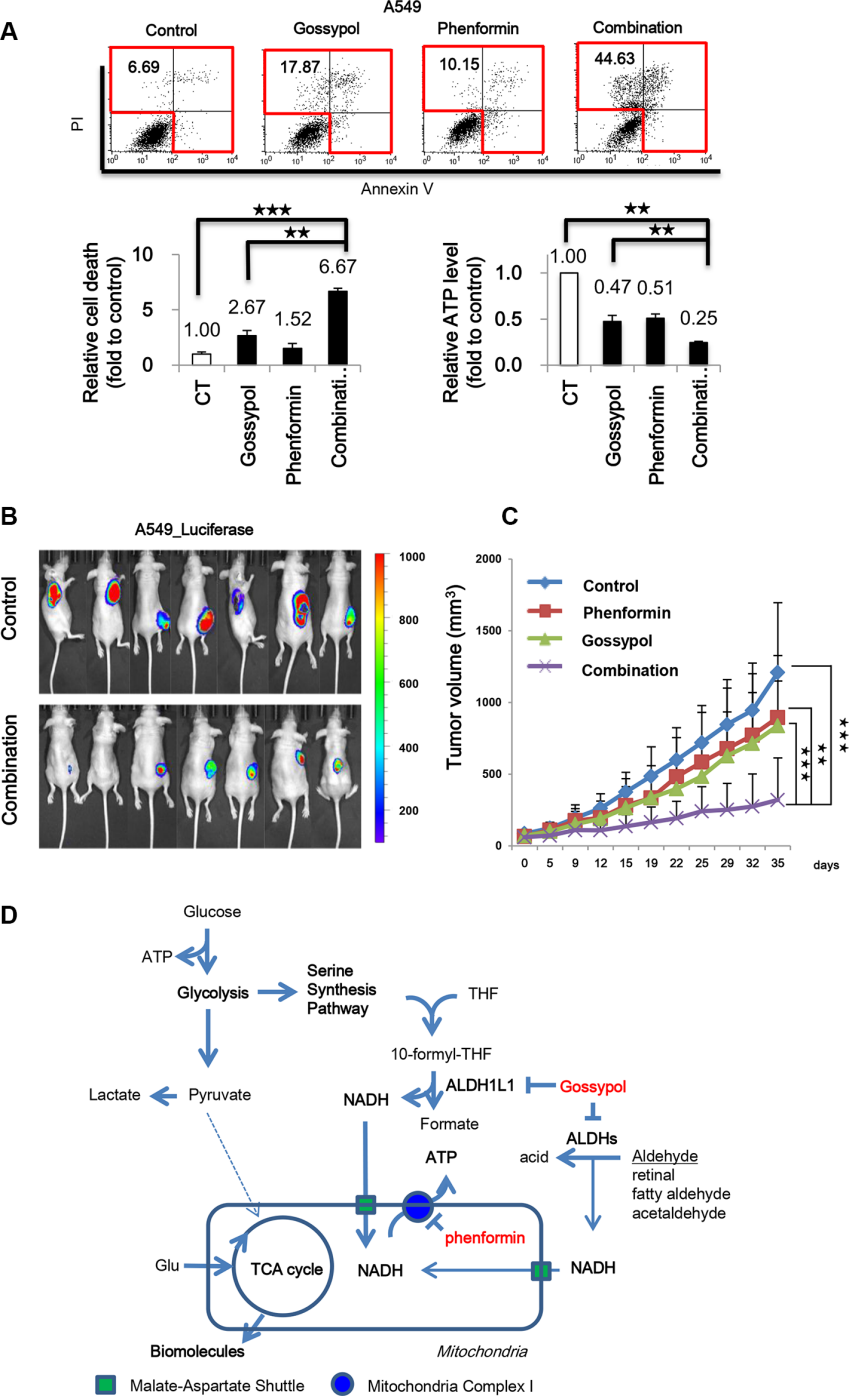
Gossypol combined with phenformin reversed NSCLC through induction of cell death via ATP depletion (**A**) Effect of gossypol (10 μM), phenformin (100 μM), or combined treatment for 48 h on cell death as determined by flow cytometric analysis and ATP production by ATP assay. (**B**) IVIS imaging of A549-luciferase xenograft tumors was taken. When the tumor mass volume reached to 100 mm^3^, the mice were treated orally 7 days/week. (*n* = 7 per each group). The dissemination of cells into lungs was monitored by IVIS (Xenogen). The data were expressed as photon flux (photons/s/cm^2^/steradian), and photon flux for each measurement is represented by a colour scale. (**C**) Tumor volumes were determined as described in Materials and Methods. (**D**) Proposed model for the role of ALDH in NSCLC metabolism. ALDH1L1 plays a key role in ATP synthesis through NADH production. NADH produced in the cytosol needs to be transported into the mitochondria by the malate-aspartate shuttle for ATP production. Data are representative of the mean and standard deviation three independent experiments. **p < 0.01, ***p < 0.001 compared to vehicle control.

To test whether ATP depletion by gossypol treatment is related with NADH depletion through the malate-aspartate shuttle, NSCLC were treated with malate after incubation with gossypol. Targeted liquid chromatography-tandem mass spectrometry (LC-MS/MS) metabolomic studies revealed that gossypol induced significant metabolic alterations in NADH and ATP production (Figure 6C). Interestingly, gossypol treatment did not lead to significant metabolic changes in TCA metabolites, indicating that the decreased ATP levels observed after gossypol treatment may not be due to a reduction in electron donors generated during the TCA cycle. Some reports showed that decrease in ATP level induced cell cycle arrest at G1/S transition as well as G2/M transition [15, 16]. Delay in mitotic progression was consistent with the more decrease of ATP production by combined treatment (Figure 7A).

### Combined treatment with gossypol and phenformin demonstrated a remarkable therapeutic response in NSCLC

The combination treatment of gossypol and phenformin for 48 h induced cell death by 6.6-fold in A549 cells compared to the control while single treatment of gossypol induced cell death by 2.6-fold in A549 cells compared to the control (Figure 7A). Moreover, ATP production was decreased by 75% in A549 cells. To test whether the effect of gossypol and phenformin combination may depend on glucose level, combination treatment was performed under low and high glucose conditions (Figure S3). No death rate change was observed under low and high glucose levels (Figure S3A, S3B), which was correlated with decrease of ATP production (Figure S3C, S3D). Total ATP level was about 20% decreased under low glucose level that was 2-fold decreased level of high glucose condition (Figure S3C). This suggests that NSCLC does not depend on glycolysis for energy production but something else.

We tested whether ALDH inhibition by treatment with gossypol and phenformin produced any synergistic therapeutic effect in the NSCLC mouse xenograft model (Figure 7B, 7C). Cultured A549 cells were injected subcutaneously near the scapulae of 8-week-old female nude BALB/c mice. Oral administration of gossypol (40 mg/kg), phenformin (100 mg/kg), and gossypol (40 mg/kg) combined with phenformin (100 mg/kg) was initiated when tumors reached a volume of 100 mm^3^ and was continued for 7 days per week. Body weight change over 10 % compared to the control was not observed in mice that received the combination treatment for 2 weeks. Single administration of gossypol or phenformin did not show any therapeutic efficacy. After 5 weeks of treatment, tumor volumes were reduced significantly with combination therapy compared to vehicle-treated control as well as single drug-treated groups while body weight remained constant, clearly demonstrating the enhanced efficacy of combined treatment *in vivo*.

## DISCUSSION

H23 and EKVX cells express high levels of ALDH1L1, and knockdown of this isozyme by siRNA treatment decreased ATP by 21–39%, respectively (Figures 3B and 4B). These results suggest that ATP production in H23 and EKVX cells depends on ALDH1L1, which may be associated with folate metabolism. Recently, folate metabolism was highlighted in that NADPH production from serine-derived one-carbon metabolism is comparable to NADPH production from the oxidative pentose phosphate pathway in cancer cells [17]. Folate metabolism is typically recognized as an important metabolic pathway producing one-carbon units for nucleic acid synthesis. However, Rabinowitz revealed that approximately 50% of NADPH production in cancer cells is related with the 10-formyl-tetrahydrofolate-pathway through methylenetetrahydrofolate dehydrogenase (MTHFD)[17]. Thus, the very next step of THF formation through oxidation of 10-formyl-THF catalysed by ALDH1L1 needs to be activated. The high ALDH1L1 expression in NSCLC revealed in our study is consistent with this hypothesis (Figure 1A, 1B). Therefore, ALDH1L1 induction may be concomitantly synchronized with induction of folate metabolism.

Although the concept of cancer metabolism was introduced over 70 years ago by Dr. Warburg [18], the source of energy and the supply mechanism in cancer tumors has not been clearly understood for two reasons. First, glucose dependency may change under various culture conditions, and second, there are multiple energy metabolism pathways depending on different nutrition sources. There is no doubt that cancer cells exhibit enhanced glycolysis compared with normal cells under normal culture conditions. In tumor cells, > 60% of the lactate produced by glycolysis from glucose is actively secreted to the extracellular milieu, and a smaller fraction of < 25 % of glycolytic flux is oxidized via oxidative phosphorylation under normoxia and high glucose conditions [19]. However, another report showed that oxidative phosphorylation is the major ATP supplier regardless of the rate of glycolysis [20]. Recent studies have revealed that the mitochondrial membrane potential of cancer cells is very active, which suggests that cancer cells undergo active oxidative phosphorylation [reviewed in [21]]. In contrast to the Warburg effect, the glycolytic contribution to the total cellular ATP supply may be negligible at < 10% of the total ATP under normoxia and high glucose conditions [20]. Only under conditions of severe hypoxia (0.1% atmospheric O_2_) or hypoglycemia (2.5 mM glucose) does glycolysis become the main ATP supplier when glucose is supplied [reviewed in [21]]. This idea was supported by an experiment that showed that antiglycolytic treatment has only a marginal effect on tumor growth inhibition [22]. This suggests that cancer cells generally depend on alternative ATP supply produced via oxidative phosphorylation. NADH can be produced by various dehydrogenases, and aldehyde dehydrogenase is an important dehydrogenase in cancer tissues because it has been reported that its levels are greatly increased in cancer tissues and cancer-initiating cells.

Gossypol is a polyphenolic compound that can be made synthetically or produced inexpensively on a very large scale by the extraction of cottonseed. Gossypol is a non-competitive inhibitor against substrate in ALDH-induced oxidation, but a strong competitive inhibitor against cofactors such as NAD^+^ [11, 23]. Gossypol single treatment (40 mg/day) during a phase I/II clinical study for metastatic breast cancer refractory to doxorubicin and paclitaxel also demonstrated limited therapeutic response [24]. Recently, a randomized study combining AT-101 (*R*-(-)-gossypol acetic acid) with docetaxel in patients with advanced NSCLC showed negligible survival advantage in the second line setting [25]. This suggests that single therapy with gossypol may not be effective to induce cancer cell death. In the safety issue, gossypol was first identified as an anti-fertility agent as a result of epidemiologic studies conducted in China in the 1950s. Clinical trials in China have found that gossypol is effective on anti-fertility by oral treatment [26]. Sperm counts usually return to normal within 3 months after termination of therapy, and men treated with gossypol have fathered normal children.

To achieve a synergistic effect on ATP depletion, we employed phenformin for the combination therapy with gossypol. The mitochondrial electron transport chain (ETC) reactions are coupled to the creation of a proton gradient across the mitochondrial inner membrane. There are three proton pumps: complexes I, III, and IV producing ATP through transmembrane proton gradient via ATP synthase. Additional electrons are delivered into the quinone pool from succinate through complex II (succinate dehydrogenase). Biguanides including phenformin are widely used for diabetes mellitus treatment which target mitochondrial complex I [27]. Targeting complex I is very efficient to block ETC reaction because complex I is a rate-limiting step for ETC [27].

In this study, we found that ALDH1L1 significantly contributes to ATP production through NADH production in NSCLC. The combined treatment of gossypol and phenformin induces cell death associated with ATP depletion (Figure 7A). These observations are consistent with a previous report showing that severe depletion of ATP to levels less than 25% of control triggers cell death [28]. Combination treatment of the ALDH inhibitor gossypol with phenformin also potentiates induction of cell death along with ATP depletion (Figure 7A). Pre-clinical trials using a human NSCLC xenograft model showed a general therapeutic response to combined treatment of phenformin and gossypol. Therefore, a combination of gossypol and phenformin provides a novel therapeutic approach for NSCLC (Figure 7D).

## MATERIALS AND METHODS

### Cell culture

All NSCLC cell lines, were obtained from the U.S. National Cancer Institute (NCI; MTA no. 2702–09). Cells were incubated at 37°C and maintained at 5% CO_2_. H23, H226, IMR-90 cell was grown in DMEM/HIGH GLUCOSE medium (SH30243.01, Hyclone, Logan, UT, USA) containing 10% FBS. Lung primary cell was airway epithelial cell basal medium (PCS-300-030, ATCC, Manassas, VA, USA) with the bronchial epithelial cell growth kit (PCS-300-040, ATCC, Masassas, VA, USA). NSCLC cells were grown in RPMI 1640 medium (SH30027.01, HyClone, Logan, UT, USA) containing 10% fetal bovine serum (FBS) (SH30070.03HI, HyClone, Logan, UT, USA), penicillin, and streptomycin. siRNA duplexes targeting human ALDH1L1 (sc-78373), DHFR (sc-37078), GOT2 (sc-60052), and MDH2 (sc-89622) (Santa Cruz Biotechnology, Santa Cruz, CA, USA) were introduced into cells using Lipofectamine^®^ 3000 (L3000015, Invitrogen, Carlsbad, CA, USA) according to the manufacturer's instructions. As negative controls, cells were incubated with Lipofectamine^®^ 3000 alone and a negative siRNA (sc-37007, sc-44230) (Santa Cruz). For ALDH overexpression, p3x FLAG-CMV-ALDH isoform constructs individually expressing ALDH1L1 and DHFR were produced by Cosmogenetech (Seoul, KOREA). Each cDNA sequence of ALDH1L1 and DHFR was obtained from NCBI. The plasmids were transfected into cells using Lipofectamine^®^ 3000 according to the manufacturer's instructions.

### Analysis of ALDH1L1 by multiple reaction monitoring mass spectrometry (MRM-MS)

Each cell lysate (30 μg in 50 μL) was mixed with 15 μL of 50 mM Tris (pH 8.0) and 10 uL of 50 mM tris-(2-carboxyethyl)-phosphine, and then incubated at 25°C for 1 h. To the reaction mixture, 20 μL of 100 mM iodoacetamide was added and further incubated for an additional 1 h in darkness. The sample was diluted 4-fold with 50 mM Tris (pH 8.0) to reduce the urea concentration to less than 1 M. Proteins in the sample were digested with TPCK-treated trypsin (1:50 for enzyme:substrate ratio; Promega, WI, USA) at 37°C for 16 h. To finish the reaction, 25 μL of 0.1% formic acid was added and the sample was simultaneously spiked with 600 fmol of tryptic digests of E. coli β-galactosidase standard (AB SCIEX, Framingham, MA, USA). The digests were desalted with a C-18 spin column (Thermo), dried via vacuum centrifugation, and stored at −25°C until use.

Selection of peptide targets of ALDH1L1: We gathered amino acid sequences of ALDH1L1 from the Uniprot database (released as of 2014.02), digested the sequences in silico with trypsin, and selected unique peptides for each ALDH isozyme.

**Table:** 

Gene Name	Target or Standard	Uniprot ID	Species	Retention Time (min)	Q1 m/z	Peptide	CE (volt)	Q3 ions	Top Q3 ion
ALDH1L1	Target	O75891	Homo sapiens (Human)	24.7	714.8483	VLEVEDSTDFFK	34.6	y3.y7.y8.y9.y10	y7

Liquid chromatography MRM-MS: Dried tryptic peptides were reconstituted with 30 μL of 5% acetonitrile/0.1% formic acid, injected with a full sample loop injection of 1 μL, and separated into a Nano cHiPLC ReproSil-Pur C18 columns (75 μm i.d × 15 cm length, pore size 120 Å, particle size 3 μm; #804-00011, Eksigent Technologies, CA, USA) pre-equilibrated with 95% Solvent A (0.1 formic acid in water) and 5% Solvent B (0.1% formic acid in acetonitrile). Peptides were eluted at a flow rate of 300 nL/min with a gradient of 5–10% Solvent B for 4 min, 10–25% for 30 min, and 25–60% for 3 min, followed by a 3-min isocratic elution with 60% Solvent B. MRM measurement was executed using QTrap5500 equipped with a nanoelectrospray ion source (AB SCIEX). The MS was operated in the positive mode with the setting values: ion spray voltage of 2100 V, curtain gas at 20 psi, nebulizer gas at 25 psi, resolution at 0.7 Da (unit resolution) for Q1/Q3, interface temperature at 150°C, and scan mass range of 300−1250 m/z. The DP and CE were set on the basis of the Skyline (version 2.5) default value. Scheduled MRM mode with detection windows of 300 seconds and cycle time of 1.5 s was used. Each sample was analyzed three times with three technical replicates.

### Quantification of MRM measurements

Skyline was applied to quantify MRM measurements by calculating peak areas of extracted ion chromatograms (XICs). To determine whether a peptide was detected in each cell line, we applied highly stringent criteria: (i) raw peak area greater than 5,000, (ii) signal to noise ratio higher than 3, and (iii) coefficient of variance lower than 25% in technical triplicates. The raw peak area values were normalized as follows. Two types of standards were used for normalization. Two peptides of human glyceraldehyde-3-phosphate dehydrogenase (Swiss-Prot accession: P04406), GALQNIIPASTGAAK and LISWYDNEFGYSNR, were used as endogenous internal standards and two peptides of E. coli β-galactosidase, IDPNAWVER and GDFQFNISR, were used as external spiked standards. All statistical data was analyzed using R software (version 2.8.1) and Excel 2010 (version 14.0, Microsoft Office).

**Table:** 

ALDH isozymes	Peptide Sequence (Q1)	Fragment ion (Q1)	Representative peptide	A549	EKVK	H322M	H23	H460	Hop62	Hop92	IMB90	Primary
ALDH1L1	VLEVEDSTDFFK	y7	Y	210455.5	182277.4	72664.4	45930.8	28234.8	N.D	N.D	N.D	N.D

### Immunoblotting and immunofluorescence staining

Cells were harvested and washed in phosphate-buffered saline (PBS) and then lysed in buffer containing 20 mM Tris-HCl (pH 7.4), 150 mM NaCl, 1% (v/v) Triton X-100, 1 mM EDTA, and protease inhibitors prior to undergoing immunoblot analysis. Anti-β-Actin antibody was purchased from Sigma-Aldrich (A5441, St. Louis, MO, USA), while anti-ALDH1L1 (ab175198) antibody was purchased from Abcam (Cambridge, UK). For immunofluorescence staining, cells were fixed with 4% (w/v) paraformaldehyde and permeabilized with 0.5% Triton X-100. The cells were then stained with anti-ALDH1L1 polyclonal antibody and Alexa Fluor 488-conjugated anti-rabbit antibody (A11008, Life Technologies, Carlsbad, CA, USA).

### Immunohistochemical staining and evaluation of ALDH1L1 with human lung cancer tissues

Tissue arrays (CC5, various human lung cancer tissues; CCN5, normal human lung tissues; *n* = 57 each case) were purchased from SuperBioChip (Seoul, Korea). Immunohistochemical staining (IHC) was performed using the UltravisionLP Detection System (Thermo Fisher Scientific Inc., Fremont, CA). Briefly, after deparaffinization of formalin-fixed, paraffin-embedded breast cancer tissues, antigen was retrieved in 10 mM citrate buffer, pH 6.0, containing 0.05% Tween 20. After ethanol fixation, the tissues were sequentially treated with 3% hydrogen peroxide and Ultra V block solution. After 1 h room-temperature incubation with ALDH1L1 antibody (EMD Millipore, Princeton, NJ, USA), the slides were washed in Tris-buffered saline including Tween 20 (TBST), incubated with primary antibody enhancer for 10 min, and exposed to horseradish peroxidase-conjugated secondary antibody for 15 min. After re-washing in TBST, the tissue slides were incubated with diaminobenzidine chromogen (Scytek Laboratories Inc, Logan, UT) and counter-stained with Mayer's hematoxylin (Dako Cytomation, Glostrup, Denmark). In the evaluation of ALHD1L1 expression, the staining intensity was scored on a 0-to-3 scale: 0, no staining of cancer cells; 1, weak staining; 2, moderate staining; 3, strong staining. In addition, the percentage of positive cells among cancer cells was scored. The two scores of intensity and positive stained-tumor cell percentage were multiplied, and the resulting value was used as expression score.

### Measurement of ATP and NADH/NAD+ levels

Total ATP levels were monitored using a CellTiter-Glo Luminescent Cell Viability Assay as per the manufacturer's instructions (G7572, Promega, Durham, NC, USA). CellTiter-Glo was added to 1 × 10^6^ cells and placed on an orbital shaker to induce cell lysis, and then the samples were read on a chemiluminescence plate reader (VICTOR light 1420; integration time of 1 s). Total NADH and NAD levels were measured using the NAD/NADH Quantitation Colorimetric Kit (K337-100, BioVision, Milpitas, CA, USA). To obtain total NAD (NADH and NAD), NADH/NAD Extraction Buffer was added to 2 × 10^5^ cells and a portion of NADH/NAD extract was heated for 30 min at 60°C to decompose NAD. The NADH and NAD extracts were converted to NADH using the NADH cycling enzyme and then read on an ELISA reader (Power wave HT, Biotek, Winooski, VT, USA).

### Measurement of mitochondrial membrane potential (Δψm)

Mitochondrial membrane potential was assessed by measuring tetramethylrodamine ester (TMRE) (ab113852, Abcam), a fluorescent probe that specifically accumulates in the chamber slide (for confocal microscopy) or 6-well plate (for flow cytometry) and then treated as indicated. Twenty minutes prior to the end of each treatment, 100 nM TMRE was added to the culture medium. Cells were washed three times with ice-cold PBS. Images were captured with a Zeiss LSM510 confocal microscope (Carl Zeiss, Oberkochen, Baden-Württemberg, Germany). Cells were also collected immediately for flow cytometric analysis of fluorescence intensity using the 585 nm (FL-2) channel.

### Pre-clinical xenograft tumor model

Balb/c-nu mice (Central Lab. Animal, Highland Heights, KY, USA) were aged 6–8 weeks before tumor induction. This study was reviewed and approved by the Institutional Animal Care and Use Committee (IACUC) of the National Cancer Center Research Institute, which is an Association for Assessment and Accreditation of Laboratory Animal Care International (AAALAC International) accredited facility that abides by the Institute of Laboratory Animal Resources guide (protocols: NCC-15-126C, NCC-15-243). A549-luc-C8 cells (5.0 × 10^6^) were inoculated subcutaneously using a 1 ml syringe. After 2 weeks, the mice were divided into four groups, namely a control group treated with vehicle only (25% ethanol in PBS, 200 μl), gossypol, phenformin, or combination-treated group. Vehicle alone and drugs (gossypol 40 mg/kg/100 μl, phenformin 100 mg/kg/100 μl, *n* = 7 per group) were administered orally once per day, 7 days/week, for 35 days. Primary tumor size was measured every 3–4 days using callipers. Tumor volume was calculated using the formula, V = (A × B^2^)/2, where V is the volume (mm^3^), A is the long diameter, and B is the short diameter.

### Imaging of cancer growth using bioluminescence

To monitor photon flux, mice were anesthetized with isoflurane inhalation, and 100 ul of D-luciferin 10 mg/ml (115144-35-9, Goldbio, St Louis, MO, USA) were injected intraperitoneally (i.p.). Bioluminescence imaging with a CCD camera (IVIS, Xenogen, Alameda, CA, USA) was initiated 5 min after injection for 5 min depending on the amount of luciferase activity. The data are expressed as photon flux (photons/s/cm^2^/steradian).

### FITC annexin V apoptosis vetection

A549 cells were treated as indicated for 48 h, washed twice in cold PBS, centrifuged at 1,400 rpm for 3 min, and then resuspended in binding buffer at a concentration of 1 × 10^6^ cells/ml. Then 100 μl of the solution (1 × 10^5^) was transferred to a 5-ml culture tube before addition of 5 μl each of annexin V-FITC and propidium iodide (PI). Cells were gently vortexed and incubated for 15 min at RT in the dark. Binding buffer (400 μl) was added to each sample before analysis by flow cytometry (BD Biosciences, San Jose, CA, USA).

### DNA FACS analysis

A549 and EKVX cells were trypsinized and fixed in 70% (vol/vol) ethanol for DNA staining. For cell cycle analysis, fixed cells were washed with PBS, and subsequently resuspended in PI/RNase staining solution (0.05 mg/mL PI, 0.1 mg/mL RNase A in PBS). FACS analysis was performed by using FACSCanto II (BD Biosciences, San Jose, CA, USA).

### Statistical analysis

Statistical analysis was performed using the Student's *t* test as appropriate.

## SUPPLEMENTARY MATERIALS FIGURES


