# Supplementary material for: Aldehyde dehydrogenase inhibition combined with phenformin treatment reversed NSCLC through ATP depletion
Source: Oncotarget. 2016 Jun 30;7(31):49397–410. doi: 10.18632/oncotarget.10354 (PMC5226516; doi:10.18632/oncotarget.10354)
Supplement: Supplementary file 1 [file oncotarget-07-49397-s001.pdf]

Aldehyde dehydrogenase inhibition combined with phenformin treatment reversed NSCLC through ATP depletion

Supplementary Materials

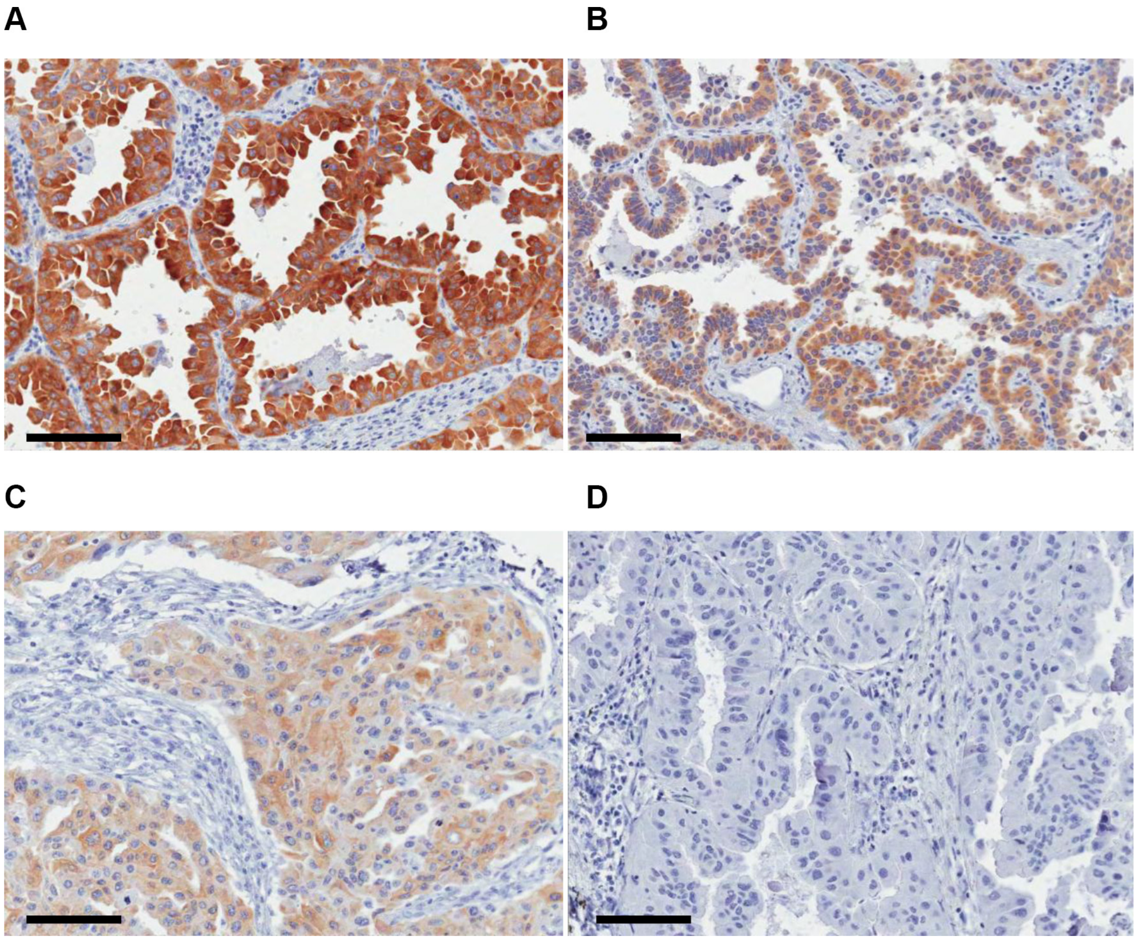

Supplementary Figure S1: Expression pattern of ALDH1L1 in lung cancer. (**A**) Strong expression (**B**) Moderate expression. (**C**) Weak expression. (**D**) Negative expression. Scale bar = 100 µm.

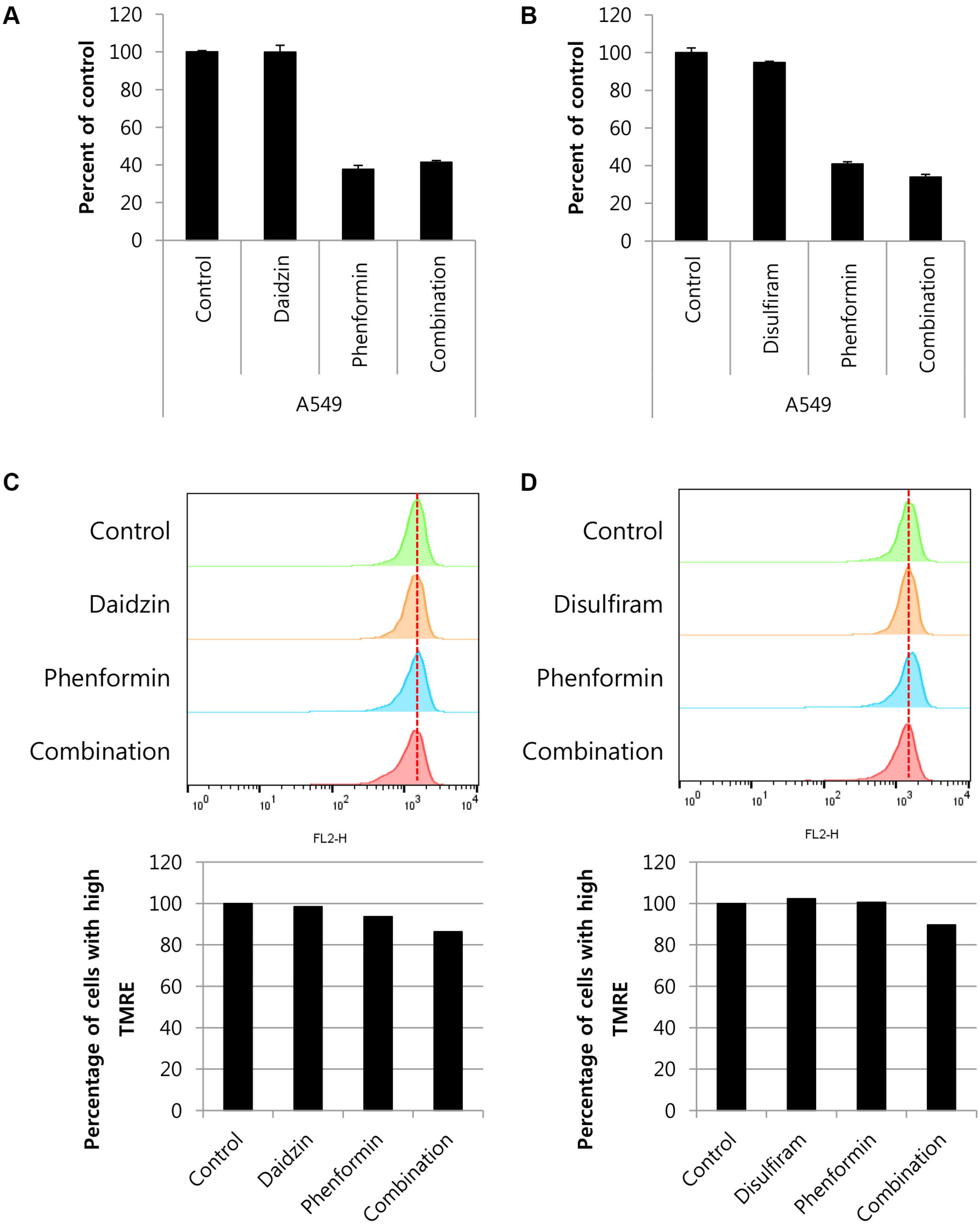

Supplementary Figure S2: Isotype specific ALDH inhibitors were not effective. (**A**) 10 µM of daidzin did not show cell growth inhibition in A549 cell by the SRB assay. (**B**) 10 µM of disulfiram also did not show cell growth inhibition. (**C**) Effect of daidzin on mitochondria potential by flow cytometric analysis was not observed. (**D**) Effect of disulfiram on mitochondria potential by flow cytometric analysis was not observed.

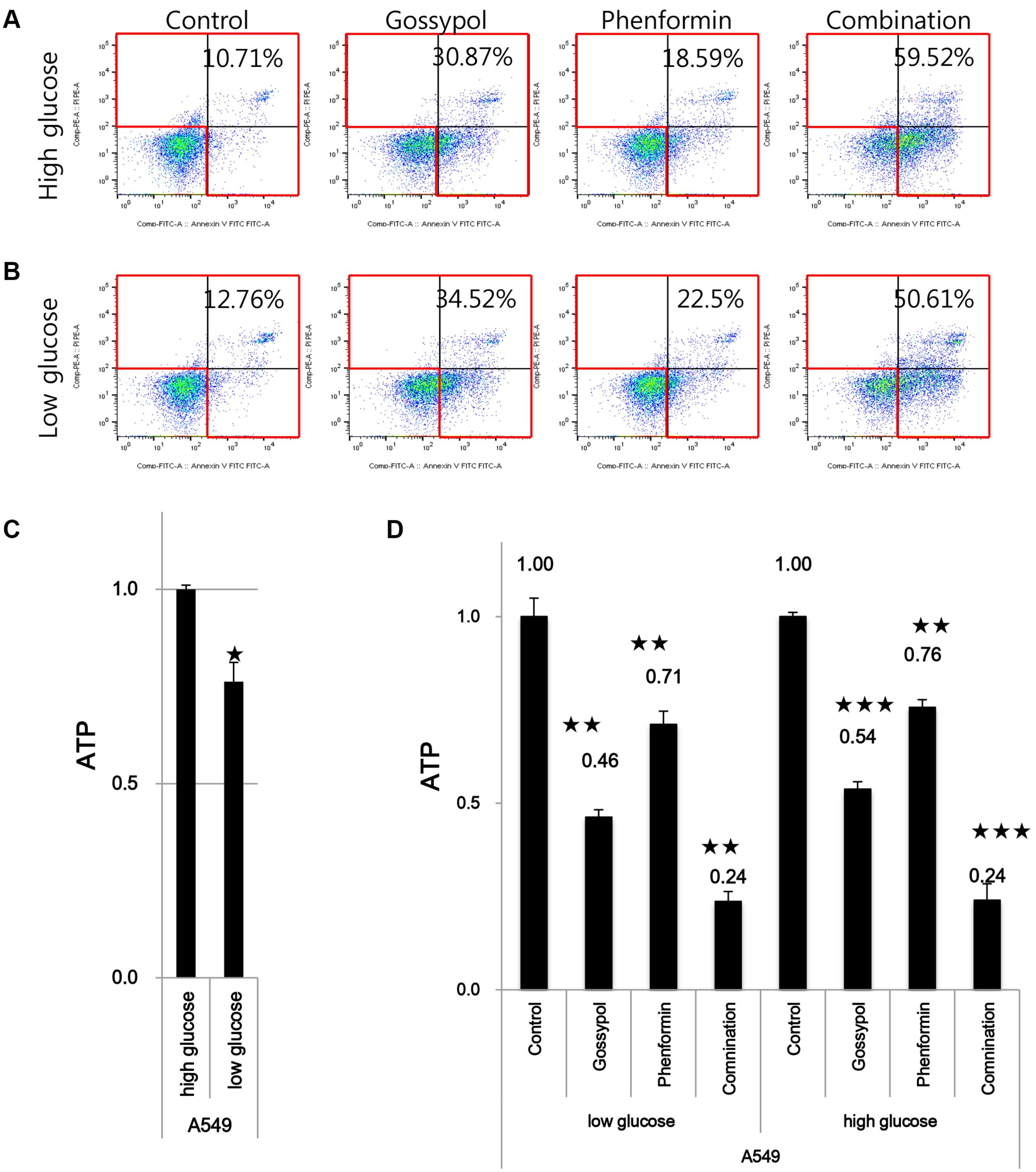

Supplementary Figure S3: Effect of gossypol, phenformin, or combined treatment for 48 h under different glucose concentrations. (**A**) Gossypol (10 µM), phenformin (100 µM), or combination was treated under high glucose (11 mM). The cell death was determined by flow cytometric analysis. (**B**) The same treatment was under low glucose (5.5 mM) condition. Cell death ratio is about the same between high and low glucose conditions. (**C**) Comparable ATP production between high and low glucose in A549 showed about 20% decrease. (**D**) The reduced productions of ATP showed about the same ratio between under low and high glucose conditions. Data are representative of the mean and standard deviation three independent experiments. ** $p < 0.01$, *** $p < 0.001$.
